# The Effectiveness of Remineralization with Compound Silver Nanoparticles and Fluoride Varnish in Carious Lesions in Primary Teeth: A Randomized Split-Mouth Clinical Trial

**DOI:** 10.3390/dj12100318

**Published:** 2024-09-30

**Authors:** María Lilia Adriana Juárez-López, Miriam Marín-Miranda, Rosita Palma-Pardínes, Raquel Retana-Ugalde

**Affiliations:** Department of Paediatric Dentistry, Faculty of Higher Studies Zaragoza, National Autonomous University of Mexico, Mexico City 04510, Mexico; miriam.marin@zaragoza.unam.mx (M.M.-M.); rositapalmapardinez@comunidad.unam.mx (R.P.-P.); retanara@unam.mx (R.R.-U.)

**Keywords:** silver-fluoride nanoparticles, cariostatic, primary teeth

## Abstract

**Background:** Anti-cariogenic properties of silver nanoparticles and fluorides have been probed mainly in vitro and with the objetive to evaluate the remineralizing effects of an applied silver nanoparticle compound plus fluoride varnish, a randomized split-mouth clinical trial was conducted in children aged 6 to 7 years. **Methods**: The project was approved by the ethics committee of the faculty. Primary molars were randomly distributed into two groups, as follows: Group A was treated with a compound based on silver nanoparticles plus fluoride varnish (SNP-FV), and Group B was treated with a silver diamine fluoride compound (SDF-KI), which is the current standard treatment. Laser fluorescence with a DIAGNOdent pen was used for the evaluation, with follow-up examinations at 15 days, 6 months, and 12 months. ANOVA test was used to compare the groups. The results showed a decrease in demineralization was observed after treatment with SNP-FV; similar results were observed after treatment with SDF-KI. Within each group, there were favorable changes. In the SDF-KI group, the differences in demineralization were 25.63 at 15 days, 29.37 at 6 months, and 30.6 at one year (*p* < 0.05). Meanwhile, in the SNP-FV group, the decreases were 22.7 at 15 days, 28.78 at 6 months, and 31.11 at one year (*p* < 0.05)**. Conclusions:** The SNP-FV combined treatment represents an alternative remineralizing treatment for the management of carious lesions in the dentin of primary molars.

## 1. Introduction

Caries is a multifactorial, noncommunicable disease that is dependent on the dental biofilm and available carbohydrate substrates. Caries results from dysbiosis and a predominance of aciduric and acidogenic microorganisms that cause demineralization, followed by the collapse of the dental structure [1,2].

The effect of dental caries on children is significant; 70% of Latin American children aged five and six years and 63% aged eleven years present carious lesions [3] In addition, low-income groups’ access to restorative care is limited, which makes managing this health problem difficult. Therefore, it is important to implement prevention strategies and alternative treatments for dental preservation. To this end, it is essential to control predisposing factors, such as dietary and hygienic habits. Moreover, the timely detection of carious lesions is important to implementing minimal-intervention strategies to stop or reverse the carious process. In addition, the histological characteristics show that caries progresses faster in primary teeth [4].

One strategy employed to stop the progression of carious lesions is the arrest and remineralization of the lesions; as such, applying cariostatic compounds constitutes an alternative, minimally invasive treatment that does not require the use of high-speed dental equipment. This treatment consists of promoting the recovery of dentin affected by caries by applying biomaterials [5].

Among the cariostatic compounds with remineralization potential are fluorides combined with silver. Silver diamine fluoride (SDF) is a solution with a basic pH that forms silver phosphate and calcium fluoride once it comes into contact with dental tissues [6]. Upon reaction with the enamel, calcium fluoride promotes the formation of fluorhydroxyapatite, while silver nitrate acts on hydroxyapatite to generate silver phosphate, which has bacteriostatic properties derived from its mechanism of protein coagulation and dentin tubule obturation [7,8]. SDF penetrates into dentin to a depth of 50 to 200 µm without adverse effects on the dental pulp [9]. A histological study reported the formation of irregular tertiary dentin, followed by a flattened odontoblastic layer, after the application of SDF [10]. At a concentration of 38%, favorable effects of SDF use have been reported, along with the only adverse effect of dental tissue pigmentation [11]. Therefore, to reduce pigmentation, the immediate topical application of potassium iodide (KI) is recommended [12].

Recently, through the use of nanotechnology, the addition of silver nanoparticles to dental products such as fluorides has been proposed [13]. Moreover, in vitro studies of such materials have indicated that they have bacteriostatic properties, and clinical trials have reported their ability to arrest carious lesions without the undesirable effect of pigmentation that is observed with SDF [14,15,16,17]. Compounds based on silver nanofluoride (SNP-F) have preventive, antimicrobial, and remineralization properties similar to those of SDF; therefore, they have been used alone and in combination with resins, ionomers, and fluorides. They can inhibit the adhesion and growth of cariogenic bacteria, preventing the demineralization of dental tissue [18,19].

Against this backdrop, it was postulated that combining a silver nanoparticle compound with a silane difluoride varnish (FV) could be a simple and low-cost alternative to conservative treatment to stop the progression of, remineralize, and control dental caries in pediatric patients; this would prove especially helpful in the treatment of children who are uncooperative due to fear or special needs, as well as those in low-income communities. However, it is first important to confirm that SNP-F can promote remineralization and the arrest of the carious process. Thus, we set out to evaluate the cariostatic effect of a silver nanoparticle compound with a difluorosilane in primary molars, determine its dental remineralization effects in carious lesions using laser fluorescence, and compare it with a compound based on SDF, which is considered the standard treatment. Our hypothesis is that combining a silver nanoparticle compound with an FV will have a similar effect to SDF.

## 2. Materials and Methods

### 2.1. Ethical Considerations and Design

A randomized split-mouth clinical trial was conducted in 40 pediatric patients aged 6 to 7 years from a primary school in an urban area of the municipality of Nezahualcóyotl. This study was approved by the Research and Bioethics Committee of the Faculty of Higher Studies Zaragoza, UNAM FESZ/CE/21-208-05 (registration number: ISRCTN 18357873).

Informed consent was requested from the parents or guardians of the participants; the children’s consent was also obtained, and all the procedures were carried out following strict biosafety measures and the principles of the Declaration of Helsinki, with a minimum level of risk for the participants.

### 2.2. Sample Size

The sample size was calculated based on the differences in treatment success rates reported in previous studies [15,20]. Assuming a difference of 37% between the groups, with a power of 80% and a standard error of 0.05%,

N = 2 (1.96 + 0.84) ^2^ (0.465) ((0.535) = 3.9 = 28

             (0.37) ^2^                                                         0.137

Because this is a longitudinal study and in order to balance possible losses, a loss of 30% was estimated: N = 28 + 9 = 37 children to include at least 74 molars for each treatment.

### 2.3. Participant Eligibility

Participants had to have at least 2 primary molars with cavitated carious lesions and no signs or symptoms of irreversible pulpitis diagnosed by spontaneous unprovoked pain or infectious processes. Bitewing and periapical X-rays were taken to exclude molars with deep cavity depths close to pulp < 0.05 mm, less than half their root length, and periapical pathological processes. Similarly, children who showed rejection behaviors and did not cooperate with the diagnostic procedures were excluded. The molars selected were randomly assigned to one of the treatments.

### 2.4. Calibration and Blinding

To determine caries activity, the operator was calibrated in advance using ICDAS criteria (Kappa 0.79). For the evaluation procedure conducted using the fluorescence method, it was not possible to blind the operators since SDF stains demineralized hard tissues, which then appear black.

### 2.5. Intervention

The participants’ cariogenic risk was determined as part of the school’s program using a questionnaire about risk factors, clinical conditions, and protective factors based on CAMBRA [21]. Oral hygiene was measured according to the presence of biofilm on dental surfaces using the O’Leary index. Oral education and supervised dental biofilm control and brushing technique advice were provided once a month. Clinical examinations of the schoolchildren were performed to select the molars with active carious lesions using the ICDAS criteria, employing visual and tactile inspections to identify surface discontinuities and textures without clinical or radiographic signs of pulp involvement or periapical pathology. The recruitment period lasted for two months and the follow-up period spanned 12 months after the first application. The molars were randomly distributed into each of the study groups by the operator, who did not participate in the evaluation process.

Group A: Silver nanoparticles plus difluorosilane varnish [22] (SNP-FV).

Group B: Silver diamine fluoride 38% plus potassium iodide [23] Riva Star (SDF-KI).

The fluorescence method was used to determine the demineralization value with a DIAGNOdent Pen 2190 [24] that was previously calibrated on a caries-free surface for each of the participants according to the manufacturer’s instructions. Later, three measurements of the carious lesions were taken, and the average of these values was recorded. The measurements were performed after cleaning and drying the selected carious lesions with a cotton swab.

**Figure 1:**
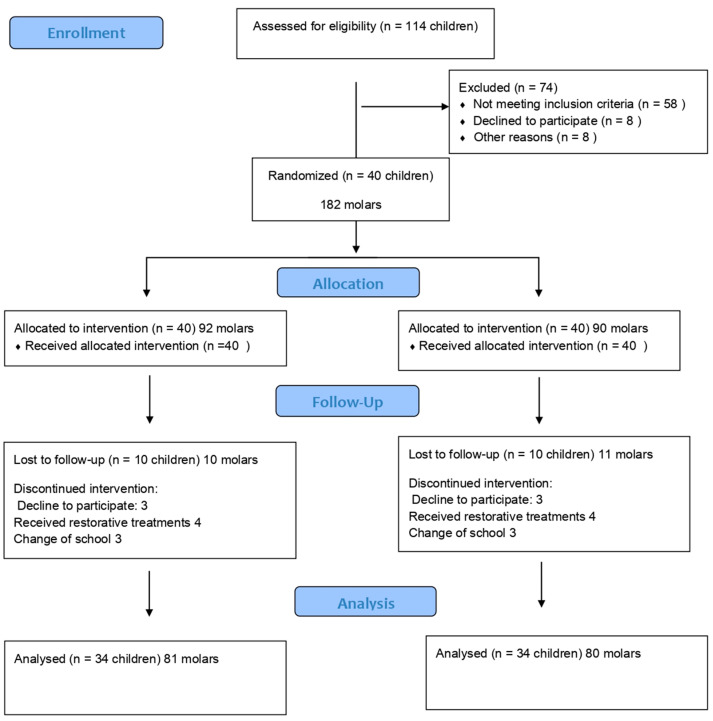
Flow diagram.

During the 12-month follow-up period, the students were given guidance on their brushing technique and advised to brush their teeth after meals. The specialist clinicians asked the participants about the presence or signs of pain and referred those cases that required other oral treatments to the University Dental Clinic. The molars that were eliminated because they were filled or exfoliated, in addition to those cases when the participant did not remain at the school, are shown in Figure 1.

### 2.6. Synthesis of the Compound Silver Nanoparticles

Silver nanoparticle powder (99.5% pure; particle size of less than 100 nanometers (0.5 g); Sigma-Aldrich CAS 576832 Saint Louis, MO, USA) was combined with polyvinylpyrrolidone (PVP; 0.02 mg/mL in water), which is commercially available in the food industry, by creating a uniform dispersion with vigorous shaking [16,25]. The mixture was prepared and stored in a cold and dry place in an amber bottle that is resistant to light. Visually, the suspension appears to be a slightly viscous light gray homogeneous liquid. When observed every month for six months, the appearance of the compound did not change, and it did not crystalize or separate. For the addition of fluoride, a second step was added during the application with 0.1% difluorosilane varnish (Fluor Protector ampules from Ivoclar Vivadent). This combination was previously tested in vitro, and it was observed to have bacteriostatic properties [26].

### 2.7. Clinical Application

The compounds were applied by specialist clinicians who did not participate in the case evaluation. Two quadrants were selected for each patient, onto which each of the treatments was randomly applied.

The procedure consisted of first washing the cavity; the superficial infected dentine was not removed and, under relative isolation with cotton rolls, each carious lesion was cleaned with a chlorhexidine swab followed by a water swab and a dry swab. The compounds were applied in two steps using a microbrush. For treatment with SDF-KI, as a first step, the SDF 38% solution was applied, followed by the application of potassium iodide (Riva Star). For the SNP-FV treatment, the nanoparticle compound was applied first, followed by the fluoride varnish Fluor Protector. In all cases, in the first step, the compound was allowed to react for two minutes, and, after the second step, another two minutes of reaction were permitted. Participants were asked not to eat or drink for one hour.

Five applications of the compounds were carried out in each group: three applications with an interval of one week in the baseline stage followed by another application at 6 months and another at 12 months.

The laser fluorescence evaluations were carried out before the intervention, 15 days after the application of the baseline treatment, and 15 days after each of the applications at 6 and 12 months. Our analysis of the results related to the decrease in the fluorescence values of the lesion. ICDAS criteria were followed after the last application.

### 2.8. Statistical Analysis

The data were analyzed using SPSS IBM V27, descriptive statistics were calculated, and ANOVA and Tukey post hoc tests were used for multiple and paired sample comparisons of the groups of interest.

## 3. Results

Initially, the participants included 40 schoolchildren, 23 girls (57%) and 17 boys (42.5%), with a total of 182 molars included in the study. Over the course of the 12-month follow-up period, 10 students withdrew: 3 due to a change in campus, 3 who decided to no longer participate in the study, and 4 who received restorative treatment. All the schoolchildren had a high level of cariogenic risk. The epidemiological data at the baseline stage are presented in Table 1. According to the ICDAS, the average number of healthy teeth was 13.6 ± 3.2, while 1.1 ± 1.4 teeth had incipient lesions, and 6.7 ± 2.7 had cavitated lesions. A total of 161 molars were included, of which 81 were subjected to treatment A, while 80 were subjected to treatment B.

**Figure 2:**
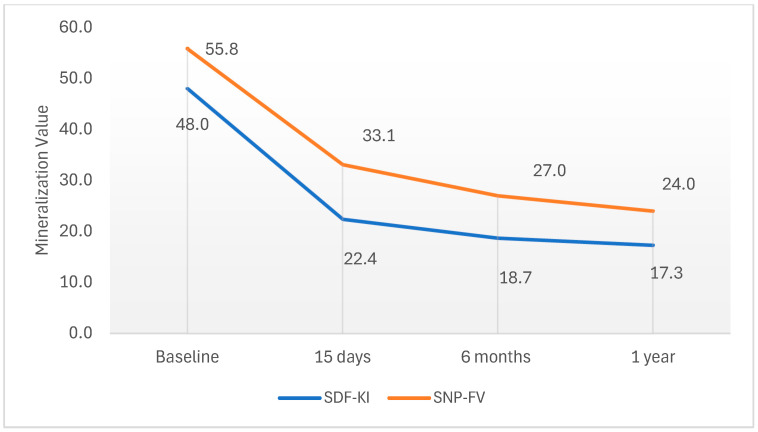
Laser fluorescence values at 15 days, 6 months, and 12 months after the application of silver diamine fluoride combined with potassium iodide and silver nanoparticles combined with fluoride varnish.

Favorable changes were observed in the demineralization values between the different treatments. Figure 2 shows that there were significant decreases in the demineralization values in the experimental and standard treatment groups (*p* < 0.05). In the SDF-KI group, the differences with the baseline values were 25.63 at 15 days (*p* < 0.05), 29.37 at six months (*p* < 0.05), and 30.6 at one year (*p* < 0.05). Meanwhile, in the SNP-FV group, the decreases were 22.7 at 15 days (*p* < 0.05), 28.78 at six months (*p* < 0.05), and 31.2 at one year (*p* < 0.05). Table 2 shows the p-values between the SNP-FV and the standard treatment in the different evaluation periods.

The SNP-FV treatment effectively remineralized carious lesions, showing no differences compared with the SDF-KI treatment.

## 4. Discussion

When managing carious lesions in pediatric patients, arresting these lesions is a minimally invasive alternative treatment; it is a low-cost method that is easy to apply. In this context, researchers have proposed using compounds based on silver nanoparticles combined with fluoride as cariostatic and remineralizing agents [27].

Silver nanoparticles are dispersions of solid particles with sizes ranging from 10 to 100 nm that can be used alone or in combination with resins, ionomers, or fluorides for the prevention of cavities [28]. Due to their bactericidal effects, silver nanoparticles have been indicated for use in restorations to minimize the microbial population and avoid the recurrence of carious lesions; moreover, it has been reported that they prevent the demineralization of enamel and dentin [29]. Although these materials’ antimicrobial properties were not the focus of this study, it should be mentioned that the inhibition of bacterial acidic products, as well as the infiltration and precipitation of silver ions in the lesion, promotes the hardening and remineralization of the lesion, since these products inhibit the demineralization caused by acidogenic bacteria. Silver ions are active against aciduric microorganisms such as Streptococci and other bacteria due to their ability to penetrate the cell wall, cause structural changes in the bacterial cell membrane, deactivate respiratory enzymes, and inhibit protein synthesis, all of which cause bacterial death [30]. In addition, the surface area for microbial contact is greater with nanoparticles, which amplifies their effects [31].

In this study, we observed that a treatment with silver nanoparticles plus silane fluoride varnish produced mineralizing effects, with a decrease in the laser fluorescence similar to that observed with SDF-KI. The two-step application of SNP-FV combines the antibacterial mechanism of silver nanoparticles with the remineralization properties of fluoride, which, in addition to interfering with microbial metabolism, promotes remineralization. A difluorosilane varnish system containing 0.1% fluoride and using ethanol and water as solvents to allow for lower viscosity increased wetting and flow. After the solvent evaporated, the fluoride concentration was up to four times higher on the tooth’s surface, providing 3% fluoride content on the applied surface [32]. Therefore, it was determined that the varnish used in this study facilitated the immediate availability of fluoride by generating calcium fluoride. Other studies have highlighted the mineralizing ability of silver nanoparticles. Silver ions have been reported to adhere to hydroxyapatite crystals and inactivate bacterial collagenases so that the preserved collagen acts as a matrix for the deposition of mineral crystals [33,34]. Silver nanofluorides have also been reported to increase the microhardness of dental tissue, [35] significantly reduce tooth demineralization, and likely increase remineralization [36]. The arrest of cariogenic activity was observed in 74% of the cases treated with SNP-FV, with an effect similar to that of SDF (79%). The results of the present study are similar to those reported by Targino et al. [13], who also observed remineralization; Santos [15], who noted that silver nano fluoride and SDF showed similar levels of effectiveness after 12 months; and Quritum [16] and Nahiredy [20], who reported that the carious process effectively stopped after the application of silver nanoparticles with fluorides. Moreover, Buitron et al. reported a higher rate of remineralization after treatment with a nanoparticle fluoride than after treatment with water [29].

In this study, 38% SDF-KI was used as a standard compound, as it inhibits matrix metalloproteinases (MMPs), which play a role in the enzymatic degradation of collagen. Previous reports have noted a decrease in the depth of the demineralized lesion and an increase in the surface microhardness after the application of SDF [37,38].

It should be noted that all the carious lesions included in the present study were mineralized after the application of SNP-FV, and the degree of demineralization decreased at the follow-up examinations at fifteen days and six and twelve months.

Additionally, slightly less pigmentation was observed after the application of the nanoparticle-based compound than after the SDF plus KI treatment, which is desirable for treatments that aim to arrest carious lesions. This observation agrees with the findings of Espindola et al., who reported very low levels of dentin staining with NSF compared with SDF based on a digital spectrophotometric investigation [39]. However, this study was limited by the fact that it did not use a color scale to determine the changes accurately. On the other hand, given that the remineralizing effect of the fluoride varnish is proportional to the fluoride concentration, the use of a higher concentration may improve the results. In the present study, which employed a randomized split-mouth methodology, the biological variables involved in the remineralization process, such as the saliva composition and dental biofilm control, as well as hygienic and nutritional habits, were equalized to avoid possible bias.

To achieve greater effectiveness in this study, three applications were made at the baseline stage and then one every six months. This scheme was based on previous studies that indicated that six-monthly applications are more effective at arresting carious lesions; additionally, initial fluoride therapy is recommended for children at a high risk of caries [7,8,17,20,27]. The follow-up period was twelve months, during which the cavities remained open. There were no symptoms of pain or infection in the treated lesions; however, it is advisable to seal or restore the lesions with a glass ionomer to obtain better clinical results. The silver nanoparticles did not cause undesirable side effects; therefore, the combination of silver nanoparticle compounds and fluoride varnish is recommended as a cariostatic and remineralizing agent, which may be useful as a noninvasive caries treatment.

## 5. Conclusions

- The present study demonstrated that SNP-FV is a mineralizing agent and a noninvasive option for the prevention and treatment of carious lesions in dentin.
- The application of SNP-FV is inexpensive, noninvasive, and simple, and it results in minimal pigmentation.

## Figures and Tables

**Table 1:** Epidemiological indices.

Index	Baseline
	X ± SD
O’Leary *	48.8 ± 21.3
DMFT ^∑^	0.11 ± 0.4
dmft ^Τ^	6.7 ± 2.3

* Oral hygienic index: the percentage of surfaces with biofilm are detected after the application of a developer die; ^∑^ Decay, missed, or filled permanent teeth; ^Τ^ Decay, missed, or filled primary teeth. X: average; SD: Standard desviation.

**Table 2:** Decreases in fluorescence values after the application of silver diamine fluoride and silver nanoparticles with a fluoride varnish.

	SDF-KI	NPS-FV	Significance
	X ± SD	X ± SD	*p*-value
Basal	48.01 ± 29.9	55.8 ± 32	0.397
15 days	22.38 ± 16.2	33.1 ± 25.8	0.068
6 months	18.64 ± 13.5	26.9 ± 21.26	0.301
1 year	17.33 ± 14.5	24.6 ± 19.1	0.710

X: average; SD: Standard desviation.

## Data Availability

The data presented in this study are available upon request from the corresponding author.
